# Calibration Framework for Modeling Nonlinear Viscoelastic–Plastic Behavior of Bioresorbable Polymers in Finite Element Analysis for Stent Applications

**DOI:** 10.3390/polym17212863

**Published:** 2025-10-27

**Authors:** Nicklas Fiedler, Thomas Kleine, Stefan Oschatz, Selina Schultz, Niels Grabow, Kerstin Lebahn

**Affiliations:** 1 Institute for Biomedical Engineering, University Medical Center Rostock, 18119 Rostock, Germanystefan.oschatz@uni-rostock.de (S.O.);; 2 Department Life, Light & Matter (LLM), University of Rostock, 18059 Rostock, Germany

**Keywords:** polymer-based biomaterial, FEA, numerical simulation, viscoelastic material model, viscoplastic material model, polymer stent

## Abstract

Finite element analysis (FEA) is common in biomedical engineering for combining design and material development, with model validation crucial for accurate prediction of material behavior. Simplified geometries are commonly needed in stent development due to high effort in prototype manufacturing. This study outlines a methodology for FEA validation related to stent development-related FEA validation using injection-molded planar 2D substructures from a stent design with two types of polymers: poly(l-lactide) (PLLA) and poly(glycolide-co-trimethylene carbonate) (PGA-co-TMC). Specimens underwent quasi-static and cyclic testing, including loading, stress relaxation, unloading, and strain recovery. The material model coefficients for FEA were calibrated for three different constitutive models: linear elastic–plastic (LEP), Parallel Rheological Framework (PRF), and Three-Network (TN) model. The validation of planar stent segment expansion (PSSE) showed strong agreement with the experiments in deformation patterns, with varying force–displacement responses. The PRF and TN models provided better fits for behavioral predictions, with the PRF model being especially favorable for PLLA, while all models exhibited limitations for PGA-co-TMC. This study proposes a robust approach for the material modeling in stent development, enabling efficient material screening and stent design optimization through a simplified 2D validation setup. Material model accuracy depends strongly on calibration–load case congruence, while phenomenological approaches (PRF) show enhanced model robustness against load case variations compared to physically coupled models (TN).

## 1. Introduction

The current study presents a constitutive material model for representative biomedically relevant thermoplastic polymers with fundamentally different material properties, poly(l-lactide) (PLLA) and poly(glycolide-co-trimethylene carbonate) (PGA-co-TMC), along with its corresponding testing methodology. Our hypothesis was that by collecting experimental data through a small number of application-specific material tests, sufficient data for advanced material models would be generated for the definition of nonlinearities, such as time dependency, viscosity, plasticity, and loading type asymmetry. Further aspects, such as fatigue or degradability, were not assessed.

The selected test protocol presented in this study to generate the material model for thermoplastic polymers is comprised of a standard uniaxial tensile test with different strain rates and a derived cyclic test with loading, unloading, and changing strain rates. In addition, as validation is often inadequately addressed in the current literature, the main objective was to provide experimental validation of the material model in the context of stent application by using planar stent segment expansion (PSSE) as an advanced test geometry for uniaxial testing. Furthermore, stress relaxation simulations should demonstrate viscoelastic and time-dependent behavior for calibrated material models, as that is not represented excessively by PSSE. In summary, the presented method can be used especially for on-demand finite element simulations during stent development, where one focus is to establish experimental setups that are designed to achieve maximum model quality over a wide range of possible material characteristics, within a manageable time frame for experiments.

Thermoplastic polymers are frequently used in biomedical engineering for the manufacture of load-bearing systems in particular vascular stents [1,2,3,4] or as a coating, with or without an incorporated drug, on different substrates [5,6].

Historically, the primary focus of polymer stent development was on cardiovascular applications, notably during the early 2010s [4]. The lessons learned from the earliest CE-certified products, in particular after the Abbott Absorb follow-up studies [7,8,9,10], such as long-term mechanical limitations through increased recoil and late lumen loss, are still being subject of ongoing scientific efforts and show continuous refinement [11]. The materials established during this period, such as biodegradable polyesters, with poly(l-lactide) as a well-established representative, remain the benchmark, primarily due to the extensive clinical data collected for these materials [4,12,13]. For the development of novel stent devices for innovative fields of application in cardiology [14], otolaryngology [15], gastroenterology [16], or interventional radiology [17] with these and derived materials, the acquisition of application-specific data is imperative.

These highly specialized applications necessitate a wide array of mechanical properties inside this polymer class. In this context, finite element analysis (FEA) is an essential tool for accelerating the development of new medical devices. FEA enables software and automation to provide targeted support for iterative development stages in design or material development [18,19,20]. However, due to the intrinsic nonlinearity of the mechanical characteristics of polymeric materials, in particular thermoplastics, the adequate modeling for in silico studies is highly multifaceted. Notably, the nonlinearity of material properties under realistic load cases is determined significantly by load amplitudes, frequencies, and surrounding media of the application site [19,21,22].

The achievable accuracy of a material model and therefore the attainable result quality are usually proportional to the complexity of the chosen constitutive model for describing the mechanical behavior. In particular, for thermoplastics, there have been numerous studies describing dependencies of the mechanical properties on temperature, molecular weight, and aging in recent years [23,24,25]. However, many existing models rely on simplified mechanical assumptions, failing to capture key aspects such as time-dependent effects, viscoelastic behavior, and plastic deformation adequately. Nevertheless, simplifications are necessary for conducting material suitability studies efficiently [20,26,27].

The most fundamental representation of the material behavior of thermoplastics is the definition of the material as a homogenous elastic material. However, in the context of FEA for stent application, this leads to limitations regarding essential stent deployment mechanics, such as elastic recoil for balloon-expandable devices. The incorporation of plasticity would increase the applicability, exemplified by the representation of plastic deformation following expansion and therefore reasonable recoil characteristics. Nevertheless, the modeling of thermoplastics remains inadequate due to non-consideration of time-dependent effects, such as strain rate dependency or relaxation, or linear approximation of elastic and plastic material behavior. In order to develop robust and application-specific material models, the key to efficiency is the collection of mechanical data that precisely defines targeted properties and neglects non-incorporated behaviors. Moreover, key aspects of biodegradable polymers, such as durability, fatigue, and degradation effects, are not incorporated, as these phenomena require specialized investigations rather than being part of an initial screening process.

With regard to this, network-based constitutive models can overcome the aforementioned limitations. The basic idea of these material models is to integrate elastic, viscous, and plastic properties and selectively superimpose them in parallel and in series. This approach enables the representation of highly intricate materials through superposition of ordinary mathematical models [2,28]. The design of the individual network arms and the description thereof using mathematical models differ noticeably. In the case of polymeric materials, experimental tests should aim to deviate from standard testing methods and instead target material behavior for the calibration of material models [1,26]. In the context of genuine loading conditions, FEA regarding structural mechanics, loading and unloading scenarios, as well as loading rates is crucial to accurately represent the diverse circumstances in the application setting of stent systems [29,30].

Different degrees of complexity and methodological approaches are described in the literature. In regard to constitutive models used, isotropic elasto-plastic models rely on experimental data obtained through uniaxial tensile test data of standardized dogbone specimens [31] or tubular specimens [32], whilst advanced approaches incorporate biaxial stretching in common and transverse directions [20] as well as different load types (tension, compression, shear, etc.) and strain rates [33]. The use of network-based constitutive models enables the combination of experiments including loading, unloading, creep, and strain rate variations [1,25,28,34,35].

**Figure 1:**
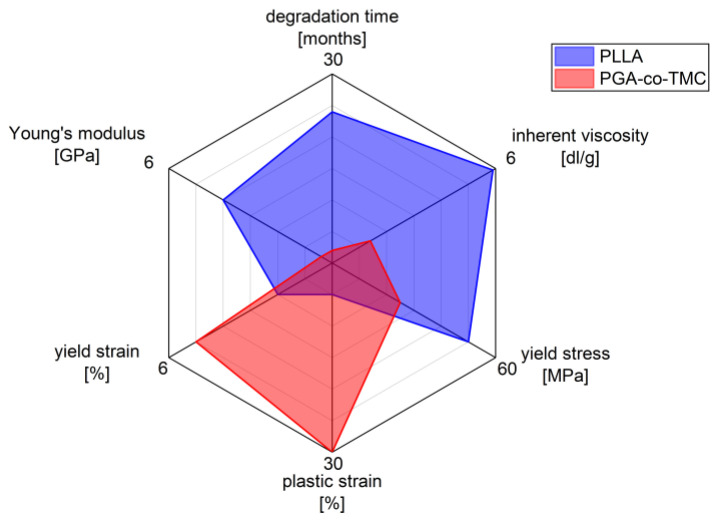
Characteristics of biodegradable polymers PLLA and PGA-co-TMC in biomedical applications, highlighting differences in mechanical and physio-chemical properties (measured data for mechanical properties stiffness, yield strain, plastic strain, and yield stress; see Section 2.1 manufacturer’s data for inherent viscosity and degradation time).

The selected polymers, PLLA and PGA-co-TMC, were chosen as clinically relevant representatives of degradable biopolymers with correspondingly different mechanical properties (Figure 1). PLLA is of particular relevance in the manufacture and application of biodegradable biomedical devices, including stents, bone screws, drug coatings, and scaffold materials [36,37]. It supports a wide range of processing techniques [38]. However, one of the key limitations of PLLA is its brittleness and low ductility, which are, among other factors, influenced by its thermal history. Additionally, its in vivo degradation time is relatively long, often exceeding the necessary period required for tissue regeneration [2,39].

The other chosen representative, polyglycolide (PGA), also exhibits inherent brittleness and a low elongation at break, but degrades rapidly in vivo, typically within several weeks [40]. However, copolymerization with trimethylene carbonate (TMC) introduces soft segments that substantially improve ductility and elasticity. This modification also allows modulation of the degradation profile, enabling clinically relevant degradation windows ranging from several weeks to months [41,42]. PGA-co-TMC copolymers are already used clinically, for example, in absorbable sutures (e.g., Maxon), and are also under investigation as drug delivery matrices [43,44] and as base material for stent applications [45].

Our findings were that robust material models for multiaxial load cases can be developed from limited uniaxial data, enabling efficient material screening and design optimization, particularly in the context of biomedical stent applications. Simpler approaches, e.g., linear elastic–plastic (LEP) material models, fail to capture key behaviors under complex loading scenarios, such as crimping or recoil. To address this, we implemented network-based viscoelastic–plastic models, which rely heavily on the match between calibration and application load cases for accuracy. Manual tuning of material coefficients and boundary conditions improved the robustness of the model. Although implicit simulations are efficient, explicit methods are necessary for capturing dynamic effects, and element selection must be made in such a way that they reflect the material behavior and avoid instabilities during FEA.

## 2. Materials and Methods

**Figure 2:**
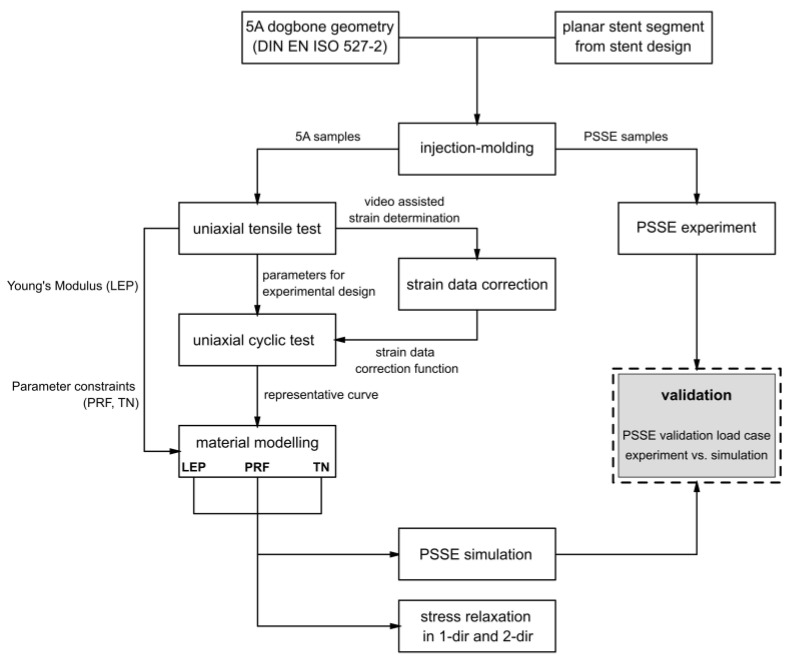
Flow chart of experimental methodology and data stream of obtained data.

The experimental design and modeling workflow to characterize and calibrate the nonlinear viscoelastic–plastic behavior of bioresorbable polymers for stent applications is visualized in Figure 2. Specimens with a standardized geometry and application-specific planar stent segments were manufactured by injection molding and subsequently subjected to uniaxial tensile testing, uniaxial cyclic testing, and PSSE experiments. Obtained strain data was corrected via a video-assisted method. The obtained testing results provided the basis for parameter identification in the material modeling process. The calibrated material parameters were integrated into FEA, followed by validation against experimental PSSE and stress relaxation analyses in one and two directions. Detailed descriptions of the testing procedures, experimental methodologies, and modeling approaches are provided in Section 2.1, Section 2.2, Section 2.3 and Section 2.4.

### 2.1. Sample Preparation

**Figure 3:**
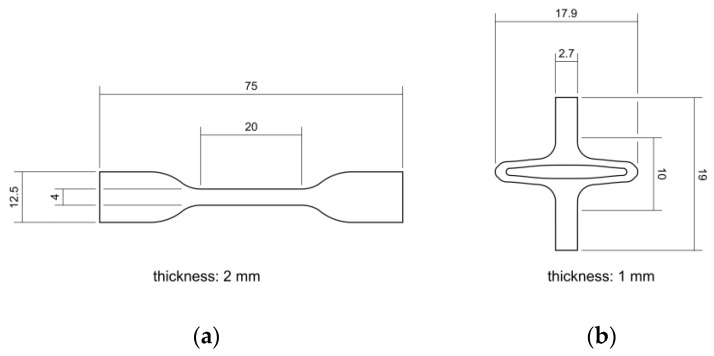
Schematic illustration of injection-molded sample geometries for quasi-static and cyclic uniaxial testing according to DIN EN ISO 527-2 [46] (**a**) and for application specific planar stent segment expansion (**b**).

PLLA (Resomer L 214 S, 5.9 dL/g, Evonik, Essen, Germany) and PGA-co-TMC (Resomer GT 643 S 1.0–1.4 dL/g, Evonik, Essen, Germany) were used after drying under reduced pressure at 40 °C for 24 h. The samples for mechanical testing were prepared by injection molding with Haake MiniJet II (Thermo Fisher Scientific, Karlsruhe, Germany). The specimen geometry 5A for uniaxial tensile tests and uniaxial cyclic testing was chosen according to DIN EN ISO 527-2 [46]. Specimens for PSSE were manufactured through injection molding with a custom-made aluminum mold generated from a stent design and scaled up by factor seven. Here, a slightly expanded design for better crimping capability after manufacturing was used [47,48]. Processing parameters for injection molding of PLLA were set to *T*_polymer_ = 230 °C, *T*_mold_ = 65 °C, *p*_injection_ = 780 bar (5 s holding time), and *p*_hold_ = 300 bar (10 s holding time). Equivalent processing parameters for PGA-co-TMC were set to *T*_polymer_ = 210 °C, *T*_mold_ = 64 °C, *p*_injection_ = 480 bar (5 s holding time), and *p*_hold_ = 300 bar (10 s holding time). For thermal equilibration, PLLA specimens were annealed at 85 °C for 60 min and PGA-co-TMC specimens at 45 °C for 15 min following the injection molding process. Figure 3 shows both sample geometries with basic dimensions.

### 2.2. Experimental Testing

#### 2.2.1. Uniaxial Tensile Testing

Uniaxial tensile tests in accordance with DIN EN ISO 527-1 [49] were performed using a universal testing machine Zwicki ZN 1.0 (ZwickRoell, Ulm, Germany) equipped with a 1.0 kN load cell. Tests were carried out at ambient temperature with crosshead speeds of 0.5 and 50 mm/min (PLLA: *n*_0.5_ = 4, *n*_50_ = 3; PGA-co-TMC: *n*_0.5_ = 5, *n*_50_ = 3). Crosshead speeds correlate with strain rates of 0.025 and 2.5 min^−1^, respectively. Young’s modulus and the yield point were determined by the 0.2% offset method.

#### 2.2.2. Uniaxial Cyclic Testing

**Figure 4:**
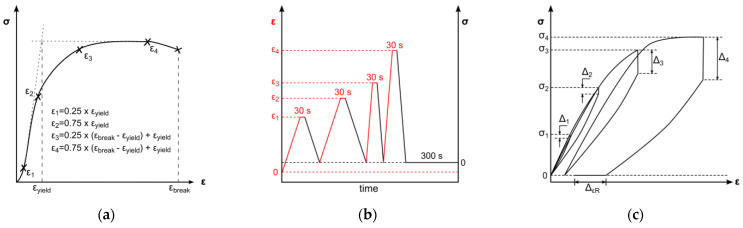
Schematic of test method for uniaxial cyclic testing. Displacement-controlled amplitudes are derived from uniaxial tensile tests (**a**). Chronological test sequences of loading, stress relaxation, and unloading with changing strain rate and subsequent recovery step. Indication of the deformation control parameter: displacement-controlled (black) and force-controlled (red) (**b**). Schematic uniaxial cyclic testing result with marked stress amplitudes *σ*_1_–*σ*_4_, derived stress relaxations ∆_1_–∆_4_, and final recovery ∆_εR_ (**c**).

An all-electric dynamic testing machine ElectroPuls E 1000 (Instron, Darmstadt, Germany) equipped with a 2 kN load cell was used for cyclic testing of 5A specimens (according to DIN EN ISO 527-2) [46]. The parameters for the test method were based on the results of one representative uniaxial tensile test. The cyclic test procedure was divided in four load cycles, composed of loading, 30 s of stress relaxation, unloading, and a subsequent 300 s recovery step. Loading steps and stress relaxations were displacement-controlled, whereas unloading and recovery were force-controlled (Figure 4b). Crosshead speeds for loading in the first two cycles were set to 0.5 mm/min and 50 mm/min for the third and fourth loading steps, respectively. The force-controlled unloading rate was equivalent to the loading rate in the elastic region of tensile testing; therefore, the average Young’s modulus of the uniaxial tests was used to determine the unloading rate (Figure 4b). Displacement-controlled strain amplitudes were set to provide data before and beyond yield point. Therefore, the strain amplitudes *ε*_1_–*ε*_4_ were defined as described in Figure 4a: two amplitudes are in the viscoelastic region, defined as 25% and 75% of yield strain, as well as two amplitudes in the plastic region, defined as 25% and 75% of strain beyond yield. The average yield point for distinct strain rates was identified by the 0.2%-offset method and resulting curve intersections. The strain beyond yield was defined according to the lowest elongation at break of all tested samples. The uniaxial cyclic tests were carried out with three specimens for each material. The quantitative evaluation of uniaxial cyclic tests was based on stress–strain characteristics. In this regard, stress amplitudes (*σ*_1_–*σ*_4_) and stress relaxations (∆_1_–∆_4_) were ascertained at every cycle’s stress relaxation strain (see Figure 4c). Furthermore, the final recovery (∆_εR_) of the procedure was determined (see Figure 4c).

#### 2.2.3. Strain Data Correction

The experimentally determined strain values from the uniaxial experiments were methodically corrected. The use of a universal testing machine in combination with dogbone specimens generated a discrepancy between the technical strain calculated from crosshead displacement and the true local strain within the specimen. By applying measurement markings that delineate the gauge length of the 5A specimens, as specified by DIN EN ISO 527-2 [46], the displacement could be accurately determined through distance analysis in individual video frames within the designated measurement region of the specimen. Video capturing was conducted with an SLR camera (Canon EOS 80D, Canon Inc., Tokio, Japan).

#### 2.2.4. Experimental Planar Stent Segment Expansion

**Figure 5:**
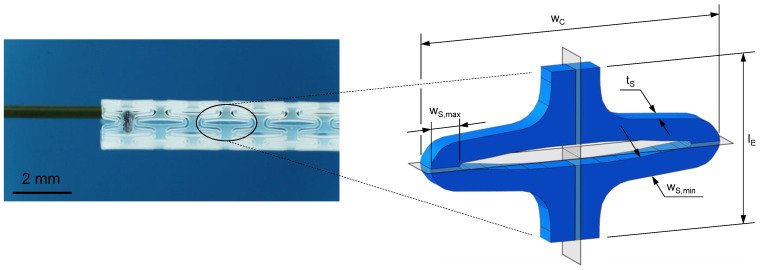
Planar stent segment geometry is derived and enrolled from a stent design previously published (**left**) [47,48]. The stent segment is a sixth of the final tubular geometry in circumferential direction with labeled dimensions segment width ($w_{C}$), strut width ($w_{S,\min}{,\;w}_{S,\max}$), strut thickness ($t_{S}$), and gauge length ($l_{E}$) (**right**).

The validation of FEA was ensured by an application-specific material test with a multiaxial load profile using examined experimental uniaxial test data for the material definition. Consequently, a segment of an enrolled stent design (Figure 5) [47,48] was manufactured via injection molding as described in Section 2.1 and uniaxially tested in a universal testing machine Zwicki ZN 1.0 (ZwickRoell, Ulm, Germany). The sample geometry (Figure 5) was scaled up by a factor of seven to facilitate sample production and handling and pronounce measuring quality. The resulting stent design has a segment width ($w_{C}$) of 17.91 mm, strut width ($w_{S,\min}$, $w_{S,\max}$) of 1.24–1.78 mm, strut thickness ($t_{S}$) of 1.50 mm, and gauge length ($l_{E}$) of 10 mm. The final displacement was set to 6.3 mm to match the radial expansion factor of the used stent design. The crosshead speed was set to 15 mm/min. After full expansion, the force was released to initiate strain recovery for 30 s.

For better comparison of experiments and simulations, video capturing for morphological analysis of different deformation states was used. Therefore, a microscope camera (Toolcraft DigiMicro 2.0 Scale, Conrad Electronics SE, Hirschau, Germany) was synchronized with the testXpert III software (ZwickRoell, Ulm, Germany) of the universal testing machine. Afterwards, a Matlab (Matlab R2024a, Mathworks, Natick, MA, USA) script using the canny edge detection operator was executed to generate contour plots for distinct time points. These time points were defined according to 5%, 15%, 25%, 65%, and 100% of final expansion and the final time point after acute strain recovery. Experimentally generated contour plots were compared with numerical simulation results. The acute strain recovery was evaluated based on the opening area of the stent segment in the fully expanded state and after unloading with following relaxation of 0.5 s (Equation (1)).

(1) $$\varepsilon_{R}=\frac{A_{\mathrm{rec}}}{A_{\exp}}-1$$

where $\varepsilon_{R}$ is the acute strain recovery, $A_{\mathrm{rec}}$ the opening area after acute strain recovery, and $A_{\exp}$ the opening area in fully expanded state.

### 2.3. Material Modeling

Material models were calibrated for the use in Abaqus 2023 (Dassault Systèmes, Vélizy-Villacoublay, France) software. As a reference material model, a linear elastic–plastic material model was calibrated directly with Abaqus CAE by using one representative uniaxial tensile test. In comparison, for representing nonlinear viscoelastic–plastic material behavior, the network-based constitutive model “Parallel Rheological Framework” (PRF), which is native to Abaqus and the “Three-network” (TN) model provided by PolyUMod (version 7.0.7, PolymerFEM, Dover, DE, USA), were investigated. The calibrations for the network-based models were performed with MCalibration software (version 7.3.1, PolymerFEM, Dover, DE, USA). Therefore, one representative cyclic test was used for the evaluation of material parameters in MCalibration due to computational efficiency. The load case was set as strain -controlled (tension only). The fitness of experimental data and material model predictions was compared by normalized mean absolute difference (NMAD).

#### 2.3.1. Linear Elastic–Plastic Material Model

**Figure 6:**
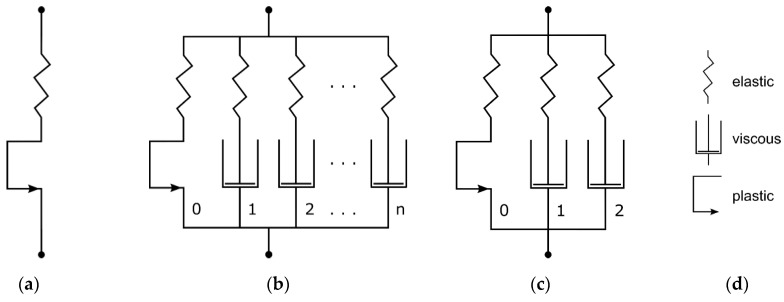
Mechanical equivalent models of linear elastic–plastic material model (**a**), generic PRF model (**b**), and Three-Network configuration with ideal plasticity in equilibrium network of PRF model (**c**). Legend for mechanical equivalent models (**d**) (modified according to [51]).

The LEP material model was defined by specifying Young’s modulus (*E*) and Poisson’s ratio (ν) for the elastic element and eight terms of stress/strain values to specify the plastic behavior ($\sigma_{\mathrm{Yn}}{/\varepsilon }_{\mathrm{Pn}}$). The mechanical equivalent model is shown in Figure 6a. Young’s modulus was estimated as the average Young’s modulus of all samples of a material examined via uniaxial tensile testing. The plastic stress/strain terms were evaluated by defining the yield stress and subsequent curve fitting through Abaqus CAE calibration features. Poisson’s ratio was set to 0.3 in accordance with the literature [31,32,50]. For the calibration of LEP material models, especially plasticity, only one representative dataset of tensile tests was used. Different strain rates or cyclic tests could not be taken into account, due to the time independence of the LEP model.

#### 2.3.2. Three-Network Parallel Rheological Framework Model

The PRF model is integrated in Abaqus 2023. It is composed of a hyperelastic equilibrium network, which can contain ideal plasticity as well. The forthcoming network arms are each defined through a scale factor referencing the equilibrium network hyperelasticity and a viscous element (Figure 6b).

Here, three PRF networks are defined as one Yeoh hyperelasticity (Equation (2))-based equilibrium network with pure plasticity (Equation (6)) and two viscoelastic networks with viscous dashpot elements (Equation (7)). This configuration (in MCalibration, “Abaqus PRF-3Net-Yeoh-Power-Plast”) is illustrated in Figure 6c. The following equations show the model’s definitions for each part of the rheological block diagram. The Yeoh form strain–energy function for modeling hyperelasticity is defined as follows [52]:

(2) $$\;U\;=\overset{3}{\underset{i=1}{\sum }}C_{i0}{\left(\bar{I_{1}}-3\right)}^{i}+\overset{2}{\underset{i=1}{\sum }}\frac{1}{D_{i}}{\left(J_{\mathrm{el}}-1\right)}^{2i}$$

where $C_{i0}$ are Yeoh coefficients, $D_{i}$ are volumetric Yeoh coefficients, $\bar{I_{1}}$ is the first strain invariant, and $J_{\mathrm{el}}$ is the elastic volume strain. The following equations define stress before and beyond yield (Equation (3)), initial yield stress $\sigma_{Y}$ (Equation (4)), and plastic stress σ beyond yield (Equation (5)) in true stress–strain curves:

(3) $$\;\sigma \;=\;\left\{\begin{array}{c} E\varepsilon \\ {\;K\varepsilon }^{n} \end{array}\right.\begin{array}{c} \sigma \;\leq {\;\sigma }_{Y}\\ \sigma \;\geq {\;\sigma }_{Y} \end{array},$$

(4) $$\sigma_{Y}{=E\varepsilon }_{Y}{=K\varepsilon }_{Y}\;\sigma \;\leq {\;\sigma }_{Y},$$

(5) $${\;\sigma =\sigma }_{Y}{\left(1+\frac{E}{\sigma_{Y}}\varepsilon_{P}\right)}^{n}\;\sigma \;\leq {\;\sigma }_{Y}$$

where σ is stress, E is Young’s modulus, K is a strength coefficient, ε is strain, n is the strain-hardening exponent, $\sigma_{Y}$ is the initial yield stress, $\varepsilon_{Y}$ is the initial yield strain. and $\varepsilon_{P}$ is the strain beyond yield. In MCalibration, the plastic material properties beyond yield are described using the power law (Equation (6)) with the following definition:

(6) $${\;\sigma \;=\;\sigma }_{Y\;}+\;\mathrm{hard}\;\cdot {\;\mathrm{PEEQ}}^{\mathrm{expn}}$$

where hard is the hardening factor, PEEQ the plastic equivalent strain, and expn the hardening coefficient. The two viscoelastic networks have a scaled Yeoh hyperelasticity of the equilibrium network. The viscous properties are defined by a power flow model (Equation (7)) [51].

(7) $${\dot{\bar{\varepsilon }}}^{\mathrm{cr}}{=\dot{\varepsilon }}_{0}{\left({\left(\frac{\tilde{q}}{q_{0}+a\;<p>}\right)}^{n}{\left[\left(m+1\right){\bar{\varepsilon }}^{\mathrm{cr}}\right]}^{m}\right)}^{\frac{1}{m+1}}$$

where ${\dot{\bar{\varepsilon }}}^{\mathrm{cr}}$ is the equivalent creep strain rate, ${\bar{\varepsilon }}^{\mathrm{cr}}$ is the equivalent creep strain, $\tilde{q}$ is the deviatoric Kirchhoff stress, p is the Kirchhoff pressure, and $q_{0}{,\;m,\;n,\;a,\;\dot{\varepsilon }}_{0}$ are material-specific parameters.

Initial values for model parameters were set automatically based on experimental data and tweaked manually in case of the yield stress ($\sigma_{Y}$) and Yeoh coefficient *C*_10_, referencing the material stiffness for small strain values according to experimental data. To facilitate the usage of the C3D8I element type for all simulations and enhance comparability, Poisson’s ratio was limited to 0.48 through definition of *D*_1_ as described in the Abaqus documentation (Equation (8)) [51].

(8) $$D_{1\;}=\frac{2}{K_{0}}=\frac{3(1-2\nu )}{\mu_{0}\left(1+\nu \right)}=\frac{3(1-2\nu )}{{2C}_{10}\left(1+\nu \right)},$$

where $K_{0}$ is the initial bulk modulus and $\mu_{0}$ the initial shear modulus, *ν* the Poisson’s ratio, and $C_{10}$ the initial shear modulus coefficient.

All coefficients were constrained during our preliminary test to enhance the efficacy and accuracy of calibration runs and avert false-positive material definitions. These were expected if the calibration of uniaxial cyclic tests was predicted to be highly accurate, but simulations of realistic multiaxial load cases demonstrated a poor prediction quality due to inconsistent material definitions.

For PLLA, 13 coefficients were selected for calibration, whereas 14 were selected for PGA-co-TMC (Table 1). For material parameter optimization, the “Covariance Matrix Adaption Evolution Strategy” (CMA-ES) followed by an “Extensive automatic search”, including an initial random search, “Levenberg-Marquardt” search, and “New Unconstrained Optimization Algorithm” (NEWUOA) search, were selected for the best fitness after at most 3000 function evaluations.

#### 2.3.3. Three-Network Model

**Figure 7:**
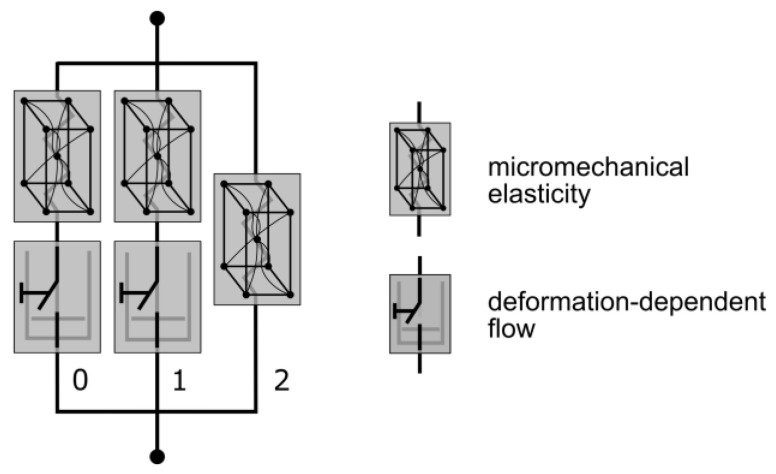
Mechanical equivalent diagram of the TN model. The elastic element is defined through a micromechanical approach and viscosity with a deformation-dependent flow rule (modified according to [54]).

The TN model is an advanced viscoelastic material model from the MCalibration/PolyUMod software package, originally developed by Bergström et al. [53]. This model is especially designed for the prediction of thermoplastic materials, due to the highly nonlinear mechanical properties. Similarly to the PRF model, the TN model is composed of three networks: one equilibrium network and two viscous networks [54]. The elastic elements are defined by Eight-Chain Models [55] and thus follow a micromechanical approach. Viscosity is addressed by Power-Flow components in each of the viscous networks (Figure 7).

The translation of these models to access Abaqus was realized via UMAT subroutines embedded by PolyUMod software.

Parameters for calibration, 9 for PLLA and 10 for PGA-co-TMC (Table 2), were initialized based on representative cyclic and static uniaxial test data. The optimization was composed of CMA-ES and a subsequent “Extensive automatic search” with a best fitness of at most 3000 function evaluations. Optimization after initialization was exclusively executed based on representative cyclic test data.

### 2.4. Finite Element Analysis

**Figure A1:**
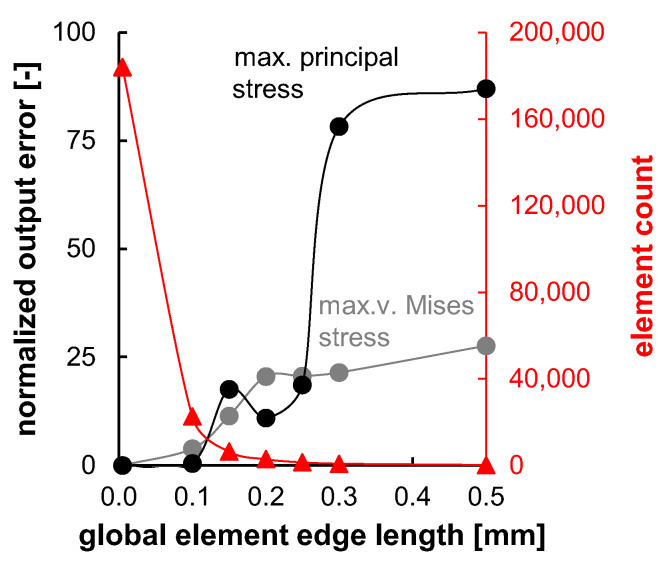
Mesh convergence studies for planar stent segment geometry. Normalized output error for v.-Mises stress and max. principal stress and element count plotted over tested global element edge lengths. The error of the smallest tested element edge length (0.05 mm) was assumed zero.

FEA was carried out using Abaqus 2023. A model for stent segment simulation was created in Abaqus CAE according to the injection-molded geometry. All simulations were conducted using the Abaqus Standard solver by applying dynamic steps. The models utilized for the numerical simulations were initialized in a stress-free, equilibrated state without intrinsic residual stresses. The element size for discretization was determined based on the results of preliminary mesh convergence studies (see Appendix A Figure A1).

#### 2.4.1. Stress Relaxation Simulation

To validate the strain rate dependence of viscosity, Young’s modulus, and (beyond) yield properties of material models in uniaxial and biaxial directions, MCalibration’s built-in virtual experiment function was used. The simulation was comprised of two segments. The initial segment delineates a constant displacement with a strain rate of 2.5 min^−1^ or 0.25 min^−1^, which corresponds to a crosshead speed of 5 mm/min or 50 mm/min for a 5A specimen geometry (according to DIN EN ISO 527-2 [46]). The second step was defined by maintaining a constant displacement for 10 s. The uniaxial tensile simulations were executed in the loading mode “uniaxial (1-direction)”, whereas the biaxial tests were conducted in the loading mode “biaxial (1- and 2-direction)”.

#### 2.4.2. Simulation of Planar Stent Segment Expansion

**Figure 8:**
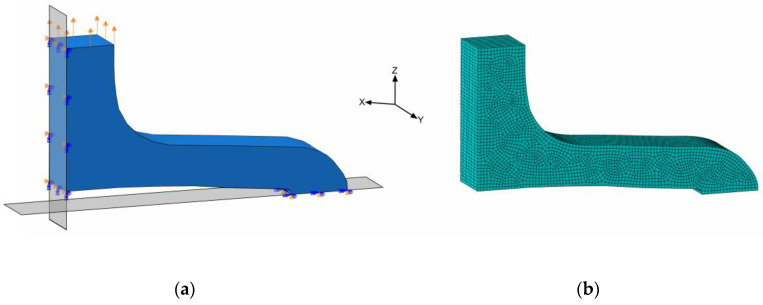
Simulation model of quarter stent segment with boundary conditions; symmetries on XY- and ZY-planes and displacement on top face (**a**) and meshed geometry (**b**).

The planar stent segment geometry described in Figure 3 and Figure 5 was simulated as a uniaxial planar expansion test. The planar extension leads to multiaxial loads in the specimens, in particular, bending. For efficiency reasons, the symmetry of the model was utilized, and only one-quarter of the stent segment was used for numerical simulations (Figure 8a).

In order to utilize the quarter geometry, symmetry boundary conditions XSYMM and ZSYMM were applied in X- and Z-directions on corresponding symmetry faces. The PSSE was implemented by a displacement boundary condition in the Z-direction at the top segment-to-segment connector face (Figure 8a). Distinct properties for the FEA regarding discretization and boundary conditions are summarized in Table 3. Here, only acute strain recovery was simulated, due to efficiency and utilization of the implicit solver.

All calibrated material models are used to simulate PSSE and are compared with experimental force–displacement data. Additionally, specimen contour morphology is analyzed at selected deformation states. As morphology is geometry-driven rather than material-dependent, only the best-fitting model from the mechanical evaluation is considered.

## 3. Results and Discussion

### 3.1. Uniaxial Tensile Testing

**Figure A2:**
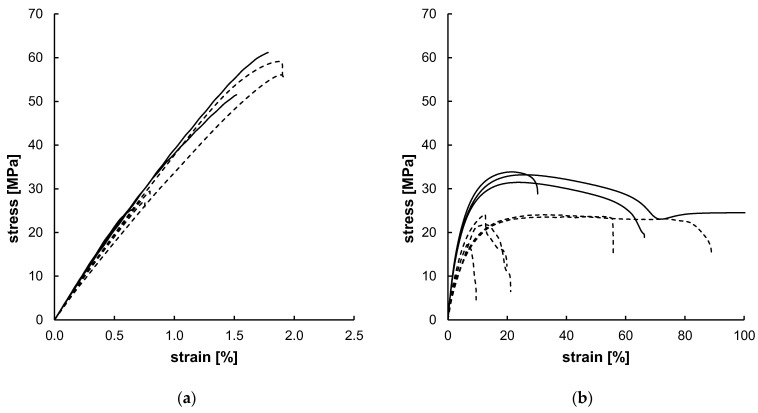
Uniaxial tensile test results for PLLA (**a**) and PGA-co-TMC (**b**) with different crosshead speeds of 0.5 mm/min (dashed; PLLA *n* = 4, PGA-co-TMC *n* = 5) and 50 mm/min (solid; PLLA *n* = 3, PGA-co-TMC *n* = 3). The highlighted sample shows an elongation at break of 832.63%, but the diagram has been limited to 100% for improved clarity.

Quasi-static uniaxial tensile tests (see Appendix A Figure A2) were analyzed following the methodology described above. Yield strain and total strain beyond yield were examined for one representative curve of each material. Examined yield strain was 1.99/3.50% (PLLA/PGA-co-TMC) and total strain beyond yield was 1.62/50.00% (PLLA/PGA-co-TMC), respectively. Resulting boundary conditions for the composition of the uniaxial cyclic experiments are summarized in Table 4. The different number of samples for both materials and strain rates is due to the limited availability caused by the challenging demolding of the samples. However, since the results of a representative curve were used and the other curves served to avoid using the values of an outlier, the number of samples did not have a decisive importance. The applied strain rates are consistent with the literature and were derived from the strain rates occurring during polymer stent expansion [1,56].

### 3.2. Uniaxial Cyclic Testing

**Figure 9:**
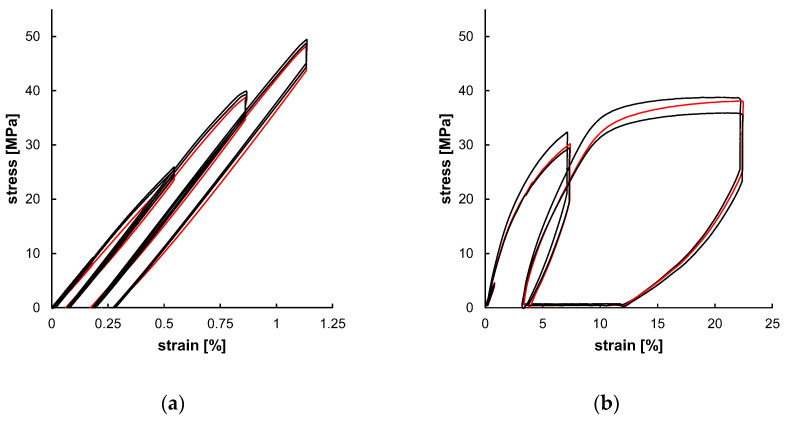
Experimental uniaxial cyclic test data for PLLA ((**a**), *n* = 3) and PGA-co-TMC ((**b**), *n* = 3) expressed as stress–strain characteristics. Red curve was used for material model optimization.

The experimental outcome of the uniaxial cyclic tests, as shown in Figure 9, served as a database for subsequent material modeling. The individually tested samples showed only minor specimen-specific deviations. Both the strain amplitudes (Table 4) were increased over the test cycles and the resulting stress amplitudes increased as well (Table 5). The resulting curves of PLLA and PGA-co-TMC differ fundamentally, primarily due to the generally limited elongation of PLLA compared to PGA-co-TMC and, in particular, the limited plasticity of PLLA. Since the plastic deformation determined in the tensile tests was used for the calculation of strain amplitudes *ε*_3_ and *ε*_4_, the cyclic tests of PLLA show only minor amounts of plasticity. The derived stress amplitudes (*σ*) and stress relaxations (Δ) are summarized in Table 5. For PLLA, the stress amplitude increased from 8.74 MPa in the first to 48.00 MPa in the fourth cycle. The stress relaxation similarly increased steadily from 0.22 MPa to 3.62 MPa during testing. Likewise, for PGA-co-TMC, stress amplitudes increased continuously from 1.60 to 34.35 MPa, while stress relaxation increased from 0.28 MPa to 10.08 MPa. This expected increase in stress amplitudes and stress relaxation in the tested strain range is caused by respective increase in strain amplitudes of the three cycles and therefore higher elastic deformation. The comparison of the final recovery revealed a stronger recovery for PGA-co-TMC (6.745%) compared to PLLA (0.042%), which is based on the more ductile material behavior of PGA-co-TMC due to the soft segments introduced by copolymerization of PGA with TMC [41].

### 3.3. Material Modeling

LEP and the network-based models PRF and TN could be generated and optimized using the described methodology. The exact definitions and material constants of the final material models’ constitution are summarized in the Appendix A Table A1 for LEP and Table A2 for PRF and TN.

**Figure 10:**
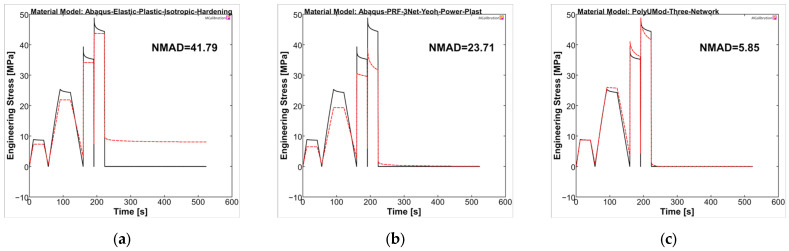
Calibrated PLLA material models ((**a**): LEP, (**b**): PRF, and (**c**): TN) in MCalibration. Comparison of representative experimental data (solid, black) and calibrated material model prediction (dashed, red) with estimated NMAD fitness.

The material models were analyzed in MCalibration regarding curve fitness of experimental data and the prediction using the generated material models. For PLLA, the NMAD was 41.79, 23.71, and 5.85 for LEP, PRF, and TN models, respectively. The LEP model showed, as expected, deficits in viscous behavior, which was addressed in stress relaxation steps of the uniaxial cyclic experiments, as well as plasticity and viscoplasticity, respectively (see Figure 10a). The PRF model was capable of reproducing viscoelastic and (visco-)plastic properties, which was evident through stress relaxations and final recovery, as also presented in the literature [35,57]. In contrast, the amplitude stresses were generally too low compared to experimental data, comparable to Karim et al. [57]. In particular, the higher correlation in the final recovery step ultimately resulted in a better NMAD value for the PRF model compared to LEP (PRF = 23.71 vs. LEP = 41.79). The TN model demonstrated the most accurate agreement between the experimental curve and the model prediction, consequently yielding the lowest NMAD value, being 5.85. It should be noted that the maximum amplitude stresses of the individual cycles and the relaxations were considerably better captured by the TN model compared to LEP and PRF, possibly due to the physical parameter approach of the TN model, rather than the phenomenological approach of the PRF model.

**Figure 11:**
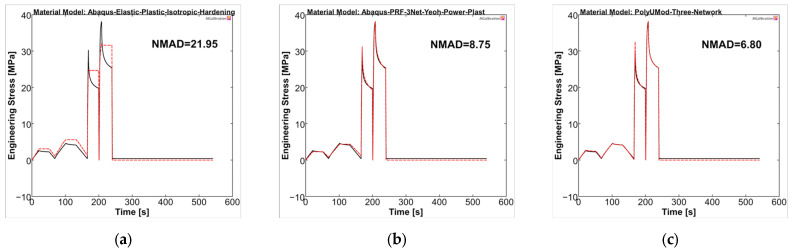
Calibrated PGA-co-TMC material models ((**a**): LEP, (**b**): PRF, and (**c**): TN) in MCalibration. Comparison of representative experimental data (solid, black) and calibrated material model prediction (dashed, red) with estimated NMAD fitness.

Overall, the selected material models demonstrated a better representation for PGA-co-TMC compared to PLLA in terms of fitness values NMAD, which were lowest for LEP (21.95) and considerably higher for PRF (8.75) and TN (6.80) (Figure 11). The stress amplitudes of the individual cycles were reproduced accurately. PRF and TN models showed very good agreement in the viscoelastic steps of the experiments, which, as expected, cannot be reproduced by the LEP model.

### 3.4. Stress Relaxation Simulation

**Figure 12:**
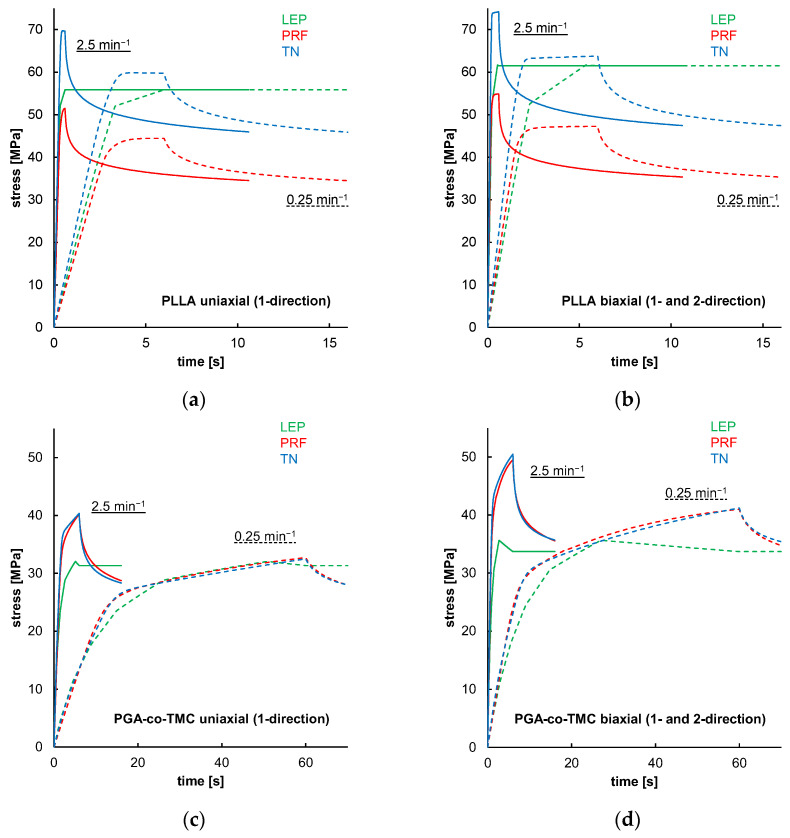
Results of uniaxial and biaxial stress relaxation simulations for PLLA (**a**,**b**) and PGA-co-TMC (**c**,**d**) using three different material models: LEP (black), PRF (red), and TN (blue). Simulations were performed at two strain rates: 0.025 min^−1^ (dashed lines) and 2.5 min^−1^ (solid lines).

Figure 12 shows simulations of one-element models that demonstrated material model behavior in the absence of geometry or model formation influences for the FEA. The results indicated that LEP models for both PLLA and PGA-co-TMC are, by definition, neither strain rate-dependent nor influenced by loading mode. In contrast, PRF and TN models showed strain rate and loading mode dependence, through diverging yield points and curve progression (Figure 12a–d). Similar findings were reported in the literature [35,53,57]. These properties are key parts of a characteristic material behavior of thermoplastic polymers in realistic loading scenarios [58].

### 3.5. Experimental and Numerical Analyses of Planar Stent Segment Expansion

The experiments of PSSE showed a limited deformation capacity for PLLA specimens, which is consistent with the results from the uniaxial tensile and uniaxial cyclic testing and the known low elongation at break of PLLA [59,60]. Therefore, the intended deformation amplitude of 6.3 mm was not reached. For comparison reasons, this data was investigated until the last frame before failure. In the case of PLLA, failure was observed at a displacement of 2.07 ± 0.31 mm (*n* = 4) for PSSE experiments until failure. Therefore, strain recovery experiments had to be adjusted to a maximal deformation of 1.5 mm for PLLA (*n* = 3) to enable acute recovery determination. Nevertheless, strain recovery after full expansion was investigated for PGA-co-TMC (*n* = 3).

The opening area of experimental data showed an increase from 18.45 ± 1.27 mm^2^ to 30.78 ± 1.90 mm^2^ (0–25% segment opening) for PLLA and 14.55 ± 1.67 mm^2^ to 54.34 ± 0.62 mm^2^ (0–100% segment opening) for PGA-co-TMC. The opening area of FEA revealed an increase from 14.53 mm^2^ to 29.73 mm^2^ for PLLA and 14.53 mm^2^ to 68.84 mm^2^ for PGA-co-TMC. Acute strain recoveries for experiments were 33.35 ± 5.54/24.28 ± 5.10% (PLLA/PGA-co-TMC) for experimental data and 23.53/33.82% (PLLA/PGA-co-TMC) for corresponding simulations. Table 6 illustrates a brief summary of segment opening and strain recovery behavior. A detailed presentation of single specimen behavior can be found in the Appendix in Table A3 and Table A4.

Ideally, the contours and, consequently, the opening area of the experimental data and the simulations would be in perfect alignment. However, the evaluation at an individual sample level and the determination of the deviations did not allow an exact assessment of the robustness of this methodological approach. Therefore, the homogeneity and reproducibility of the injection-molded samples are a critical factor in understanding the variations in the opening areas. It is crucial to note the variation in the initial opening area of PLLA and PGA-co-TMC samples, which were fabricated using the same injection mold. It can be postulated that the process of demolding, in combination with the potential for deformations during this step, resulted in irreversible deformations of PLLA samples, whereas PGA-co-TMC exhibited a less pronounced degree of deformation. A comprehensive study of this process step is necessary to provide insight and facilitate the identification of necessary modifications. Additionally, the processing-induced influence on the molecular structure, such as molecular orientation or crystallinity, which directly affects the mechanical properties of a material, should be taken into account [2,61].

**Figure 13:**
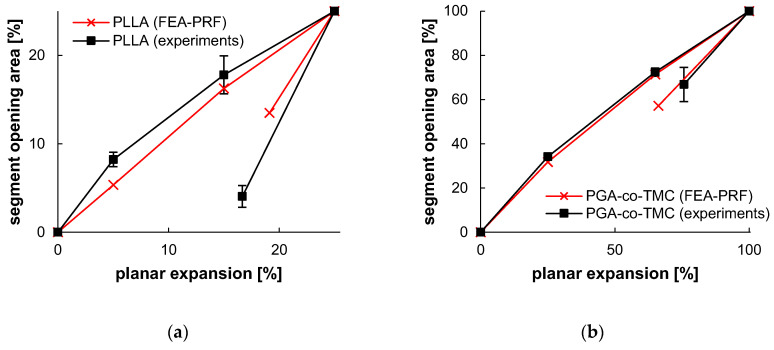
Segment opening and recovery behavior of PLLA (**a**) and PGA-co-TMC (**b**). Relative opening area, as determined from extracted contour plots (Table 6), plotted as a function of relative planar expansion.

In the context of stent development, accompanying simulations, and therefore production of a validation specimen, complexity, and processing time are key to effective and comprehensive material screening. While deviations are expected with the presented approach, they can be mitigated to a good level, as shown in Table 6, which highlights a strong correlation between experiment and simulation. The application of PSSE provides a substantially closer approximation to the realistic load scenario in stents than standard samples, for example, in uniaxial tests. The manufacture and testing of cylindrical specimens with a designated stent design, on the other hand, would rapidly increase the expense of specimen production. Yet, the deviations observed in the data indicated the presence of genuine scatter in the individual samples, thereby raising the prospect of obtaining valid and robust results when constructing material models. Overall, the plots of segment opening versus planar expansion demonstrated a high degree of agreement in the load range, with an increase in deviation in the acute recovery step, particularly at the final point of the investigation. These deviations are also illustrated in Figure 13.

**Figure 14:**
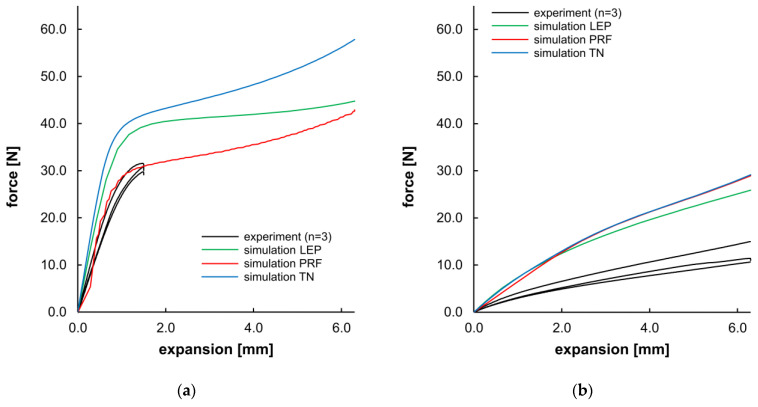
Planar expansion behavior of stent segments for PLLA (**a**) and PGA-co-TMC (**b**). Expansion force evolution plotted as a function of planar expansion.

Apart from the morphological evaluation of the opening and recovery behavior, Figure 14 presents a direct comparison of the force–displacement curves from experiments and the corresponding simulation. For PLLA, the compliance of experimental data and PRF was given until the 25% expansion level (~1.5 mm) of the target expansion. LEP and TN material models showed a higher slope in the viscoelastic domain and a higher yield stress (see Table 7). As the simulations were executed without incorporating a damage or failure model, all simulations for PLLA demonstrated a maximum expansion of 6.3 mm, despite failure at 25% in the experiments. For PGA-co-TMC, a considerably higher dispersion was observed in the individual experimental measurements as well as in the material model fitness. The agreement between material models and experimental data was overall less accurate. PRF and TN models shared a comparable curve progression, whereas LEP was slightly diverging for PGA-co-TMC. Overall, all material models for PGA-co-TMC overestimate the stiffness in this load case. For the PRF model of PLLA, higher fluctuations are visible in the curve shape. This behavior could be attributed to the fact that the material model operates close to the limit of numerical stability under the given loading conditions. During material model calibration, several parameters were constrained with physically known parameter bounds. Consequently, these parameters exhibit a narrow range for permissible results and potentially increase the sensitivity for distinct deformation states, and, in some cases, lead to local instabilities or numerical perturbations in general.

The quantitative evaluation of Figure 14 (Table 7) shows considerable scatter among individual samples’ NMAD values for evaluation of the curve fitness of experimental and simulated data. The obtained stiffness and force values were in better agreement for PLLA than for PGA-co-TMC and exhibited a consistent tendency regarding aspects of NMAD, stiffness, and the force at maximum segment opening. In contrast, the PRF model provided the best agreement regarding stiffness for PGA-co-TMC, while the LEP model is more accurate for the maximum force. These quantitative measures are consistent with the curve intersection behavior illustrated in Figure 14.

With regard to the outcomes of the material model calibration, the simulation results demonstrated pronounced variations, particularly those with network-based material models TN for PLLA and PRF/TN for PGA-co-TMC, which appeared improbable given the good NMAD fitness. However, it is important to note that the quality of the material model calibration is intrinsically bound to the initial data used for its calibration. Consequently, the primary challenge is to impose lower and upper limits on the material coefficients (in addition to the default ones), ensuring that only realistic material definitions are established. These definitions must then be implemented in diverse load cases by leveraging a minimal amount of data in 1–2 loading modes. This objective has been accomplished with great efficacy through the implementation of the PRF model for PLLA. However, minor instabilities were discernible in the curve progression of the initial area.

Conversely, the optimized load case could also be represented with a high degree of accuracy for PGA-co-TMC. Pronounced deviations were observed if variations in loading mode occurred, here PSSE. These findings suggested a more pronounced differentiation in the volumetric mechanical properties, indicating that the selected parameter limits were more effective for PLLA than for PGA-co-TMC. This could be related to the molecular orientation induced by the injection molding process, which also shows differences for homopolymers and copolymers due to their molecular structure.

The use of implicit simulations provides efficiency of validations and material screening applications. For highly dynamic processes, such as elastic recoil after stent deployment, explicit simulations must be considered. For materials from the PolyUMod library, the C3D8I element type is recommended. On the other hand, PRF calibrations in this study tended to result in material models with Poisson’s ratios greater than 0.48 without further modification, supporting the utilization of C3D8H elements to compensate for material instabilities due to incompressibility, which lead to locking effects in other element types. However, this would limit the comparability of the simulations. These limitations would also benefit from an expanded material dataset, especially data from dynamic mechanical analysis regarding viscoelastic and time-dependent aspects, including Poisson’s ratio.

In comparison with extant studies [27,62,63], our study simplifies the simulation accompanying development to a 2D level. This simplification inherently carries the potential for increased deviations. However, our findings indicate that, under properly constrained conditions, accurate three-dimensional simulations can be achieved.

## 4. Conclusions

This study evaluated a methodology for robust material models for multiaxial load cases based on uniaxial experimental data. This approach contributes to efficient material screening and design optimization in a biomedical context, particularly regarding stent development, through the utilization of a simplified 2D-validation geometry derived from a stent design.

It is important to note that simpler material models, such as LEP definitions, struggle in addressing multiaxial and time-dependent considerations with thermoplastic polymers. These models fail to capture essential material characteristics necessary for representing stent-related load scenarios, such as crimping, radial expansion, or elastic recoil. Furthermore, using these models for material screening is typically limited to a single loading scenario, particularly concerning the loading mode and strain rate.

To overcome these limitations, this study incorporated network-based viscoelastic–plastic material models. The first model, designated as the PRF material model, is directly integrated with Abaqus CAE. The second model, referred to as the TN material model, is integrated through the utilization of UMAT subroutines. The precision of these models is highly dependent on the correspondence between the load case used during model calibration and the application load case. Consequently, the NMAD fitness value obtained during the calibration of material models should be considered specific to the distinct load case. This point is relevant when looking at load cases that require the estimation of volumetric damping. When using limited uniaxial experimental data, manually adjusting material coefficients and defining realistic limits of those coefficients drastically improves the resulting material model robustness against load case deviations [64].

In conclusion, the suggested approach facilitates an effective and reliable methodology for material selection and design optimization in biomedical engineering. The results demonstrate that phenomenological modeling frameworks, such as the PRF model, exhibit a superior performance when discrepancies exist between the experimental calibration data and the validation load case. In contrast, the TN model achieves better agreement under calibrated loading conditions but shows a pronounced loss of predictive accuracy when applied to deviating or more complex load scenarios. This behavior is directly linked to its physically coupled formulation, which may limit its generalization capabilities beyond the calibration load case. In summary, the proposed framework supports informed decision-making in the development of optimized materials and structural designs in biomedical engineering, which in turn supports the development of new stent technologies.

## Figures and Tables

**Table 1:** Coefficients for material model calibration of PRF model for PLLA and PGA-co-TMC.

Material	PRF Coefficients	
PLLA	Yeoh hyperelasticity viscoelastic networks (*N* = 2) plasticity	*C*_10_, *C*_20_ *, *C*_30_ * S_1_, *q*_1_, *n*_1_, *m*_1_, *S*_2_, *q*_2_, *n*_2_, *m*_2_ σ_Y0_, hard, expn
PGA-co-TMC	Yeoh hyperelasticity viscoelastic networks (*N* = 2) plasticity	*C*_10_, *C*_20_ *, *C*_30_ * S_1_, *q*_1_, *n*_1_, *m*_1_, $\dot{\varepsilon }$_1_, *S*_2_, *q*_2_, *n*_2_, *m*_2_, $\dot{\varepsilon }$_2_ σ_Y0_, hard, expn

* Parameters were defined according to the assumption $C_{10}{\;>>\;|C}_{20}{|\;>>\;C}_{30}$ and were not further calibrated afterwards [52].

**Table 2:** Coefficients for material model calibration of TN model for PLLA and PGA-co-TMC.

Material	TN Coefficients	
PLLA	experimental data network A network B network C temperature dependence	*λ*_L_ *, *κ* *, *a* *, *q* * µ_A_, $\hat{\tau }$_A_, *m*_A_ *µ*_Bi_, *µ*_Bf_, *β*, $\hat{\tau }$_B_, *m*_B_ *µ*_C_ $\hat{\theta }$ *, *n* *, α *, *θ* *
PGA-co-TMC	experimental data network A network B network C temperature dependence	*λ*_L_, *κ* *, *a* *, *q* * µ_A_, $\hat{\tau }$_A_, *m*_A_ *µ*_Bi_, *µ*_Bf_, *β*, $\hat{\tau }$_B_, *m*_B_ *µ*_C_ $\hat{\theta }$ *, *n* *, *α* *, *θ* *

* Parameters were defined through an initial guess and according to the developers recommendations and were not calibrated afterwards [54].

**Table 3:** FEA properties of in silico PSSE simulations.

Parameter	Value
solver	Abaqus Standard
model geometry	planar stent segment
step time	-
expansion	25.2 s
acute strain recovery	0.5 s
element type	C3D8I
mesh	30,450 elements 0.1 mm mesh edge length curvature control enabled (0.05)
number of equations	499,530 (LEP), 496,080 (PRF), 496,080 (TN)
applied displacement	3.15 mm

**Table 4:** Results of uniaxial tensile tests and derived parameters for uniaxial cyclic experiments.

Parameter	PLLA	PGA-co-TMC
strain amplitudes	-	-
*ε*_1_	0.1 mm	0.08 mm
*ε*_2_	0.3 mm	0.24 mm
*ε*_3_	0.475 mm	2.74 mm
*ε*_4_	0.625 mm	7.58 mm
unloading rate	-	-
$\dot{\varepsilon }$ _0.5_	5.167 N/s	0.887 N/s
$\dot{\varepsilon }$ _50_	516.667 N/s	135.33 N/s

**Table 5:** Quality parameters derived from uniaxial cyclic charts of PLLA and PGA-co-TMC.

Material	Stress Amplitude [MPa]				Stress Relaxation [MPa]				Recovery [%]
	$\sigma_{1}$	$\sigma_{2}$	$\sigma_{3}$	$\sigma_{4}$	$\Delta_{1}$	$\Delta_{2}$	$\Delta_{3}$	$\Delta_{4}$	$\Delta_{\mathrm{eR}}$
PLLA	8.92 ± 0.48	25.43 ± 0.61	37.88 ± 0.39	48.18 ± 0.56	0.22 ± 0.01	1.01 ± 0.03	2.49 ± 0.13	3.62 ± 0.03	0.042 ± 0.001
PGA-co-TMC	2.11 ± 0.51	4.30 ± 0.28	28.34 ± 1.11	34.86 ± 1.09	0.28 ± 0.07	0.43 ± 0.03	8.19 ± 0.35	10.08 ± 0.09	6.745 ± 0.140

**Table 6:** Selected PSSE frames, extracted contour plots and FEA results for representative PLLA and PGA-co-TMC specimens at distinct expansion states and after acute strain recovery; expansion states correspond to a target segment opening of 100% at 6.3 mm displacement, with maximum achievable expansion of 1.5 mm for PLLA and 6.3 mm for PGA-co-TMC.

	PLLA				PGA-co-TMC			
	5%	15%	Max. Expansion	Acute Strain Recovery	25%	65%	Max. Expansion	Acute Strain Recovery
video frame	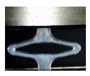	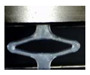	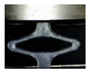	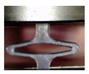	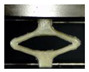	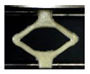	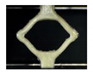	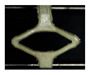
contour	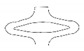	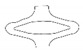	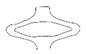	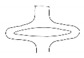	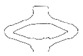	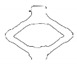	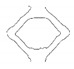	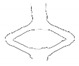
opening area [mm^2^]	20.87	25.98	29.94	18.66	30.03	44.68	53.94	38.92
v. Mises stress	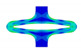	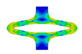	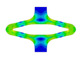	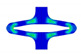	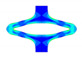	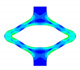	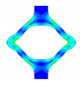	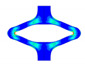
opening area [mm^2^]	17.78	24.41	29.73	22.73	31.79	53.26	68.84	45.56

**Table 7:** Quantitative examination of NMAD, stiffness, and force at maximal segment opening for PSSE experiments and simulations.

	PLLA					PGA-co-TMC				
	NMAD			Stiffness [N/mm]	F_1.5mm_ [N]	NMAD			Stiffness [N/mm]	F_6.3mm_ [N]
	PLLA-1	PLLA-2	PLLA-3			PGA-co-TMC-1	PGA-co-TMC-2	PGA-co-TMC-3		
LEP	0.41	0.45	0.28	46.38	39.36	3.28	6.00	5.08	8.34	25.90
PRF	0.17	0.20	0.07	41.24	31.14	3.73	6.62	5.64	6.37	28.95
TN	0.57	0.61	0.42	55.00	41.86	3.58	6.78	5.79	7.87	29.18
experiments				30.53 ± 3.61	29.79 ± 0.56				4.00 ± 0.70	11.91 ± 2.12

## Data Availability

The original contributions presented in this study are included in the article. Further inquiries can be directed to the corresponding author.

## Abbreviations

The following abbreviations are used in this manuscript:

C3D8H	8-node linear brick element, hybrid with constant pressure
C3D8I	8-node linear brick element, incompatible modes
CMA-ES	Covariance Matrix Adaption Evolution Strategy
FEA	Finite Element Analysis
LEP	Linear Elastic–Plastic
NEWUOA	New Unconstrained Optimization Algorithm
NMAD	Normalized Mean Absolute Difference
PGA	Polyglycolide
PGA-co-TMC	Poly(glycolide-co-trimethylene carbonate)
PLLA	Poly(l-lactide)
PRF	Parallel Rheological Framework
PSSE	Planar Stent Segment Expansion
TMC	Trimethylene Carbonate
TN	Three-Network
UMAT	User Material

## Appendix A

**Table A1:** Calibrated material model coefficients for LEP material models.

Coefficient	PLLA	PGA-co-TMC
*E* [MPa] *	4042.80	734.19
*ν*	0.3	0.3
*σ*_Y0_ [MPa]	28.00	4.54
*ε*_P0_	0	0
*σ*_Y1_ [MPa]	32.90	4.64
*ε*_P1_	6.07 × 10^−5^	4.97 × 10^−6^
*σ*_Y2_ [MPa]	37.80	11.32
*ε*_P2_	1.49 × 10^−4^	0.0028
*σ*_Y3_ [MPa]	42.69	18.00
*ε*_P3_	3.16 × 10^−4^	0.01
*σ*_Y4_ [MPa]	47.59	24.68
*ε*_P4_	5.37 × 10^−4^	0.027
*σ*_Y5_ [MPa]	52.48	31.37
*ε*_P5_	7.97 × 10^−4^	0.06
*σ*_Y6_ [MPa]	57.38	38.05
*ε*_P6_	1.16 × 10^−2^	0.14
*σ*_Y7_ [MPa]	62.27	40.75
*ε*_P7_	2.26 × 10^−2^	0.41

* Young’s modulus was defined according to averaged results of uniaxial tensile tests.

**Table A2:** Calibrated material model coefficients for PRF (MCalibration and Abaqus input-file syntax) and TN model.

PRF					TN		
Coefficient	MCalibration Syntax		Abaqus Syntax		Coefficient	PLLA	PGA-co-TMC
	PLLA	PGA-co-TMC	PLLA	PGA-co-TMC			
*C*_10_ [MPa]	4.00	0.7	603.99	91.18	*µ*_A_ [MPa]	835.99	81.97
*S*_1_	50.00	113.23	0.33	0.87	$\hat{\theta }$ [K]	0 *	0 *
*q*_1_ [MPa]	31.40	54.25	31.40	54.25	*λ*_L_	5.10 *	9.48
*n*_1_	9.31	6.50	9.31	6.50	*κ* [MPa]	6918.60 *	1018.49 *
*m*_1_	−0.09	−0.15	−0.09	−0.15	$\hat{\tau }$_A_ [MPa]	35.21	19.65
$\dot{\varepsilon }$_1_ [s^−1^]	1 *	1.07	1 *	1.07	*a*	0 *	0 *
*S*_2_	100.00	16.02	0.66	0.12	*m*_A_	10.51	2.51
*q*_2_ [MPa]	35.1747	724.97	20.00	724.97	*n*	0 *	0 *
*n*_2_	22.23	1.98	22.23	1.98	*µ*_Bi_ [MPa]	787.21	126.46
*m*_2_	−1.85 × 10^−5^	−0.5	−1.85 × 10^−5^	−0.5	*µ*_Bf_ [MPa]	783.46	33.21
$\dot{\varepsilon }$_2_ [s^−1^]	1 *	22.46	1 *	22.46	*β*	0.69	30.41
σ_Y0_ [MPa]	27.56	5.77	27.56	5.77	$\hat{\tau }$_B_ [MPa]	34.43	9.39
*hard* [MPa]	9.42	−1.19	-	-	*m*_B_	21.67	21.88
*expn*	0.94	1.58	-	-	*µ*_C_ [MPa]	17.51	21.88
-	-	-	-	-	*q*	0 *	0 *
-	-	-	-	-	*α* [1/K]	0 *	0 *
-	-	-	-	-	*θ* [K]	293 *	293 *

* These parameters were not optimized during material model calibration, as recommended by MCalibration. An initial guess based on experimental data was used instead. Parameters C_20,[1–3]_, C_30,[1–3]_, κ_2,[1–3]_, κ_3,[1–3]_, and p_0,[1–3]_ were all zero for the TN model according to MCalibration’s recommendations based on experimental data.

**Table A3:** PSSE screenshots, extracted contour plots and FEA results for PLLA specimens at distinct expansion states and after acute strain recovery. The expansion states refer to the target segment opening of 100% at 6.3 mm displacement. Maximal possible expansion without failure for PLLA is 1.5 mm.

		0%	5%	15%	Max. Expansion	Acute Strain Recovery
PLLA-1	video frame					
	contour					
	opening area [mm^2^]	19.42	23.69	28.27	33.41	21.10
PLLA-2	video frame					
	contour					
	opening area [mm^2^]	16.66	20.87	25.98	29.97	18.66
PLLA-3	video frame					
	contour					
	opening area [mm^2^]	19.28	22.97	27.42	28.99	21.59
FEA	v. Mises stress					
	opening area [mm^2^]	14.53	17.78	24.41	29.73	22.73

**Table A4:** PSSE screenshots, extracted contour plots and FEA results for PGA-co-TMC specimens at distinct expansion states and after acute strain recovery. The expansion states refer to the target segment opening of 100% at 6.3 mm displacement. Maximal possible expansion without failure for PGA-co-TMC is 6.3 mm.

		0%	25%	65%	Max. Expansion	Acute Strain Recovery
PGA-co-TMC-1	video frame					
	contour					
	opening area [mm^2^]	16.91	30.03	44.68	53.94	38.92
PGA-co-TMC-2	video frame					
	contour					
	opening area [mm^2^]	13.27	27.03	42.45	53.85	44.66
PGA-co-TMC-3	video frame					
	contour					
	opening area [mm^2^]	13.47	27.39	42.94	55.22	39.80
FEA	v. Mises stress					
	opening area [mm^2^]	14.53	31.79	53.26	68.84	45.56
